# Ageing and anaesthetic sensitivity: the role of inflammaging in the vulnerable surgical brain

**DOI:** 10.3389/fmed.2026.1920909

**Published:** 2026-10-30

**Authors:** Catalina Díaz-Papapietro, Darío Ríos, Rodrigo Brisso, Rodrigo Gutiérrez, José Ignacio Egaña, Antonello Penna

**Affiliations:** 1 Department of Anaesthesiology and Perioperative Medicine, Hospital Clínico Universidad de Chile, Faculty of Medicine, Universidad de Chile, Santiago, Chile; 2 Centro de Investigación Clínica Avanzada (CICA), Hospital Clínico Universidad de Chile, Faculty of Medicine, Universidad de Chile, Santiago, Chile; 3 Faculty of Medicine, School of Medicine, Universidad de Chile, Santiago, Chile

**Keywords:** ageing, anaesthetic sensitivity, blood–brain barrier, electroencephalography, inflammaging, macrophages, neuroinflammation

## Abstract

Ageing surgical patients demonstrate increased vulnerability to anaesthetic agents, including heightened sensitivity, reduced drug requirements, and delayed emergence, and they are also at increased risk of postoperative complications such as delirium and haemodynamic instability. These phenomena are only partially understood and have traditionally been attributed to the global functional decline associated with ageing. However, emerging preclinical and clinical evidence suggests that inflammaging and related neuroimmune alterations, including peripheral macrophage activation, blood-brain barrier dysfunction, and immune-to-brain signalling, may also modulate anaesthetic response. This narrative review examines current evidence on anaesthetic sensitivity in older adults, the role of macrophage phenotypes in ageing, and the mechanisms by which neuroinflammation may alter neuronal excitability, synaptic function, and electroencephalographic markers of anaesthetic depth. It also reviews methodological approaches ranging from rodent models to biomarkers such as the cerebrospinal fluid to plasma albumin ratio and EEG-derived indices. Overall, the available literature supports a biologically plausible link between age-related immune dysregulation and altered anaesthetic sensitivity, while also suggesting that these same pathways may contribute to broader perioperative brain vulnerability, including postoperative delirium. Direct clinical evidence remains limited, however, and many mechanistic links continue to rely on extrapolation from preclinical models. Further integrative perioperative studies combining immunological profiling, neurophysiological monitoring, and pharmacodynamic modelling are needed to clarify how neuroimmune ageing influences anaesthetic brain sensitivity in older adults.

## 1 Introduction

More than 300 million surgical procedures are estimated to be performed worldwide each year, underscoring the global scale of perioperative care (1, 2). A significant challenge for perioperative medicine is the rising number of operations performed on older adults, a rapidly growing demographic (3). Ageing is associated with a decline in physiological reserve and increased vulnerability to anaesthetic agents, including heightened sensitivity, reduced drug requirements, and prolonged emergence (4). Older adults are also at increased risk of several postoperative complications affecting multiple organ systems, including neurological (perioperative neurocognitive disorders and postoperative delirium), cardiovascular (haemodynamic instability), and respiratory (atelectasis) complications, among others (5, 6).

While these clinical observations are well recognised, the mechanisms underlying altered anaesthetic sensitivity in older adults remain poorly defined. The hypnotic component of general anaesthesia can be produced by chemically diverse agents that act through distinct molecular targets and neural circuits rather than through a single uniform mechanism (7, 8). Given the neuroimmune focus of this review, we place particular emphasis on GABA_A_ receptor-mediated hypnotics, providing a plausible link between inflammation and altered anaesthetic pharmacodynamics (9). Recent evidence has implicated age-related chronic inflammation, known as inflammaging, and associated neuroimmune alterations, such as peripheral macrophage activation and blood–brain barrier (BBB) dysfunction, as potential modulators of anaesthetic response (10–12). However, the translational relevance of these findings in humans remains uncertain.

This narrative review aims to explore the interplay between inflammaging, macrophage phenotypes, and anaesthetic sensitivity, drawing upon preclinical and clinical evidence to highlight immunological pathways that may underlie altered anaesthetic requirements in older adults. We first review the clinical features of anaesthetic sensitivity in older adults, then examine the role of inflammaging, macrophage phenotypes, and BBB dysfunction in neuroimmune vulnerability, and finally discuss the available clinical evidence, related perioperative brain outcomes, plausible interventions and remaining translational gaps.

## 2 Ageing and anaesthetic pharmacology: distinguishing pharmacokinetic from pharmacodynamic changes

The reduced anaesthetic requirements observed with advancing age arise from changes in both pharmacokinetics (PK) and pharmacodynamics (PD), which should be considered as related but conceptually distinct processes (13–15). Pharmacokinetics describes the relationship between an administered dose and the resulting plasma or effect-site concentration, whereas pharmacodynamics describes the relationship between drug concentration at its site of action and the resulting clinical or neurophysiological effect (13, 14).

Ageing produces several physiological changes relevant to anaesthetic PK (13–16). Reduced total body water and lean body mass, together with a relative increase in adipose tissue, alter drug distribution, while reductions in hepatic mass, hepatic blood flow, renal perfusion, and glomerular filtration may decrease drug clearance (13–15). Rapid distribution into highly perfused tissues may also become slower with age, whereas the apparent volume of distribution and elimination half-life of lipophilic drugs may increase (13, 14). Collectively, these changes mean that older adults frequently require smaller bolus doses and lower maintenance infusion rates to achieve plasma concentrations comparable with those in younger adults (14, 15). Thus, explaining part of the apparent reduction in anaesthetic requirements.

Conventional pharmacokinetic changes can explain how ageing modifies drug exposure, but they do not fully explain why a given brain concentration may produce a greater anaesthetic effect, as ageing also alters the concentration–effect relationship itself (13–15, 17). Older adults may exhibit more profound clinical and neurophysiological effects at a given plasma or effect-site concentration, indicating a genuine pharmacodynamic shift (14). Proposed mechanisms include age-related changes in GABA_A_, NMDA, and opioid receptor function, loss of neuronal synapses, altered intracellular calcium homeostasis, cortical structural changes, and reduced functional connectivity (14, 15). Consistent with this concept, the minimum alveolar concentration of volatile anaesthetics progressively decreases with age (18, 19), while contemporary PK–PD models demonstrate age-dependent reductions in the effect-site concentrations required to produce defined electroencephalographic effects with intravenous agents such as propofol and remifentanil (20, 21). These observations indicate that age-related reductions in anaesthetic requirement cannot be attributed solely to altered drug disposition (14, 20, 21).

The unresolved question is therefore not simply why older adults require lower doses, but what biological processes alter the concentration–effect relationship itself. We propose that age-related neuroimmune changes may represent one such pharmacodynamic modifier. Specifically, inflammaging and altered immune-to-brain signalling can modify neuronal and synaptic function, while experimental inflammation has been shown to increase neuronal and behavioural sensitivity to general anaesthetics (9, 10, 12). These observations raise the possibility that neuroimmune ageing could shift cerebral responsiveness to anaesthetic agents independently of changes in drug concentration. Whether this neuroimmune phenotype contributes directly to the increased pharmacodynamic sensitivity of the ageing human brain remains an important and testable hypothesis (9).

## 3 Anaesthetic sensitivity in older adults

The effects of general anaesthetics can be assessed clinically by the progressive loss of responsiveness to verbal, tactile, and noxious stimuli. Their effects can also be evaluated neurophysiologically using the electroencephalogram (EEG), including changes in the power of specific frequency bands and the use of processed indices derived from the EEG signal, such as the bispectral index (BIS), to estimate the depth of unconsciousness (22). At deeper levels of anaesthesia, particularly in the context of overdosage, EEG activity may evolve into burst suppression (BS), a pattern characterised by alternating periods of high-voltage activity and electrical quiescence that reflects profound cortical depression (23, 24).

Ageing is associated with a well-documented reduction in anaesthetic requirements (25); in the context of this review, this clinical observation underscores the increased neuropharmacological sensitivity and vulnerability of the ageing brain. In pharmacodynamic terms, increased sensitivity refers to a greater clinical or neurophysiological effect at a given drug concentration. The minimum alveolar concentration (MAC) of volatile agents—defined as the alveolar concentration that prevents a motor response to a standard surgical stimulus in 50% of patients—decreases by approximately 6% per decade after the age of 40 (19). Similarly, intravenous agents such as propofol also exhibit increased sensitivity in older adults, reflected in lower doses required for loss of consciousness (LOC) (26–29) and greater EEG evidence of hypnotic effect at lower doses (22, 30).

Recent studies using processed EEG indices such as the BIS and spectral entropy have shown that older adults may reach deeper anaesthetic states at lower drug concentrations than younger counterparts (31, 32). In this context, BS is particularly relevant; as an electroencephalographic pattern associated with excessive anaesthetic depth, it serves as a marker of relative overdosage—indicating that a standard anaesthetic dose has become disproportionately high for a vulnerable or ageing brain. Older patients appear to show age-related increases in the duration and prevalence of intraoperative BS, suggesting greater susceptibility to excessive cortical depression under standard dosing strategies (4, 33–35).

In recent years, increasing attention has been directed towards the influence of age-related immune and neuroinflammatory changes on anaesthetic sensitivity. A growing body of evidence suggests that chronic low-grade inflammation, termed “inflammaging”, can alter neuronal function and synaptic plasticity (10, 36–38). Furthermore, immune-to-brain signalling may become dysregulated with age, leading to enhanced neuroinflammation, glial priming, and compromised BBB integrity (39–42). These changes may modulate central nervous system (CNS) responses to anaesthetic agents through mechanisms such as altered GABAergic tone, receptor desensitisation, and impaired neuronal homeostasis (43–45). Preclinical models have also shown that systemic inflammation can increase anaesthetic sensitivity independently of drug metabolism (9).

Thus, while conventional pharmacological models provide a partial explanation, the contribution of neuroimmune modulation and BBB dysfunction to anaesthetic sensitivity in older adults requires further investigation.

## 4 General anaesthetic targets: the GABA_A_ receptor

Increased sensitivity, strictly speaking, is a pharmacodynamic phenomenon, namely a greater effect at a given concentration. Consequently, pharmacodynamic factors, including age-related changes in neuronal responsiveness to general anaesthetics and the possible influence of systemic immune signalling, must also contribute (46–49). Hence, the GABA_A_ receptor emerges as a plausible pharmacodynamic substrate of age-related changes in anaesthetic sensitivity, as it represents a molecular target for several routine anaesthetic agents.

Propofol, etomidate and volatile anaesthetics exert their hypnotic effects through positive allosteric modulation of GABA_A_ receptors (7, 50, 51). Accordingly, the influence of ageing and inflammation on anaesthetic sensitivity is likely to depend on the drug, molecular target, neural circuit, and pharmacodynamic endpoint being examined.

Avramescu et al. showed that inflammatory signalling increased neuronal GABA_A_ receptor surface expression, enhanced GABAergic responses in the presence of etomidate and isoflurane, and increased hypnotic sensitivity to these agents *in vivo* (9). Importantly, inflammatory effects differed according to the anaesthetic and endpoint assessed, supporting the concept that neuroimmune modulation of anaesthetic sensitivity is agent- and circuit-specific rather than uniform (9). We therefore place particular emphasis on GABA_A_ receptor-mediated hypnotics, while recognizing that the effects of neuroimmune ageing on other anaesthetic classes require separate investigation.

## 5 Hallmarks of ageing: inflammaging and immune–brain crosstalk

Ageing is increasingly understood through the framework of the hallmarks of ageing, a set of interconnected biological processes that drive the functional decline and increased disease susceptibility observed in older adults (52). These hallmarks underpin many age-related conditions, including cardiovascular, neurodegenerative, and metabolic disorders (53–57). Among them, inflammaging—the chronic, sterile, low-grade inflammation that accompanies ageing—has gained recognition as a key contributor to immune dysfunction and brain vulnerability in the perioperative setting.

Inflammaging has emerged as a key paradigm for understanding how the aged immune system influences perioperative vulnerability, particularly within the brain (10, 52). This persistent inflammatory state arises from multiple converging mechanisms, including cellular senescence, immune cell reprogramming, gut dysbiosis, and mitochondrial dysfunction, which collectively sustain a pro-inflammatory milieu (52, 58, 59).

A hallmark feature of inflammaging is the persistent activation of monocytes and macrophages, which acquire pro-inflammatory phenotypes and contribute to elevated systemic levels of cytokines such as TNF-α, IL-1β, and IL-6 (60). These mediators have been associated with broader perioperative brain vulnerability, including adverse cognitive outcomes in older surgical patients (11, 61–63). While other innate immune cells, such as neutrophils and dendritic cells, also undergo age-related dysfunction, macrophages play a pivotal role due to their longevity, plasticity, and tissue-resident sentry functions (64–66). During ageing, this balance shifts towards a primed, hyperresponsive macrophage state, characterised by exaggerated responses to minimal stimuli and impaired resolution of inflammation (67, 68).

Macrophage-driven inflammaging extends to the CNS through complex immune-to-brain signalling pathways. These include diffusion of cytokines across a compromised BBB, cell trafficking, and activation of endothelial and glial cells (69, 70). Aged macrophages also contribute to the senescence-associated secretory phenotype (SASP), which includes pro-inflammatory cytokines, chemokines, and matrix-remodelling enzymes that perpetuate neuroinflammation (68, 71). These signals amplify the activation of astrocytes and microglia, drive oxidative stress, and impair synaptic plasticity, particularly in regions critical for consciousness and cognition (72, 73).

### 5.1 Peripheral macrophage signalling and BBB permeability

The BBB, once considered an immunoprivileged interface, becomes increasingly permeable with ageing and systemic inflammation (74–76). Age-related changes in endothelial cells, including tight junction disruption, altered transporter function, and increased transcytosis, facilitate the passage of peripheral immune mediators and cells into the CNS (77). Monocyte-derived macrophages, in particular, can infiltrate the perivascular space and brain parenchyma, further fuelling neuroinflammation (72).

Peripheral macrophage-associated inflammation may influence brain function through several complementary immune-to-brain signalling pathways that do not necessarily require direct macrophage entry into the CNS (12, 78). Circulating cytokines, chemokines, complement components, and other inflammatory mediators contribute to the systemic inflammatory milieu of ageing (79). Recent evidence further implicates complement signalling as a potential link between macrophage ageing and neural vulnerability. Mishra et al. showed that circulating C3a increases with age in humans and mice and identified an age-associated, non-senescent macrophage subset in visceral adipose tissue as a major source of C3a. In these macrophages, autocrine C3a–C3aR1 signalling through ERK promoted age-related inflammation, whereas C3a neutralisation attenuated inflammaging in mice, supporting complement activation as an active component of macrophage-associated immune ageing. At the CNS level, Crowley et al. demonstrated that neuronal hyperactivity induces region-specific, C1q-dependent synapse loss in the adult hippocampus, establishing a mechanistic connection between complement activation and activity-dependent synaptic remodelling. Although neither study directly examined anaesthetic sensitivity, together these findings raise the hypothesis that complement dysregulation may provide an additional pathway linking peripheral immune ageing with synaptic vulnerability and altered neuronal responsiveness to anaesthetic agents (79, 80). Among these signals, circulating cytokines can be sensed by the cerebral endothelium, which should be regarded not merely as a passive barrier but as an active component of the neurovascular unit capable of translating systemic immune signals into local endothelial and inflammatory responses (41, 77, 78). Age-related endothelial dysfunction, altered transcytosis, changes in tight-junction integrity, and dysregulated adhesion and chemotactic signalling may further amplify this communication and, under conditions of greater barrier dysfunction, facilitate leukocyte trafficking into perivascular and parenchymal compartments (41). At the brain borders, perivascular and other border-associated macrophages are strategically positioned to detect circulating signals and interact with the neurovascular compartment, providing an additional interface between peripheral and central immunity (81, 82). These pathways may promote or amplify microglial and astrocytic inflammatory responses, leading to local cytokine production and alterations in synaptic plasticity and neuronal function (72, 73, 83). Importantly, whether microglial priming is itself a causal mediator of altered anaesthetic sensitivity or instead reflects a pre-existing state of age-related brain vulnerability remains unresolved (9, 83). We therefore propose that ageing-related immune dysregulation may modify anaesthetic pharmacodynamics through a multistep process linking peripheral macrophage activation, neurovascular signalling, CNS immune amplification, and ultimately changes in neuronal excitability and network responsiveness to anaesthetic agents (9).

Moreover, inflammatory cytokines such as IL-6, TNF-α, and IL-1β modulate GABAergic and glutamatergic signalling, thereby affecting neurophysiological pathways that are directly relevant to the action of many anaesthetic agents (84). Preclinical models further suggest that peripheral macrophage infiltration can impair cortical arousal and alter EEG correlates of anaesthetic depth, including reduced alpha power and BS (85–87).

While direct human evidence linking macrophage phenotypes to anaesthetic sensitivity remains scarce, the convergence of ageing, inflammaging, and neuroimmune vulnerability provide a biologically plausible framework that requires further translational research. Anaesthesia research would benefit from integrating multimodal data: immune profiling, markers of BBB permeability—such as the cerebrospinal fluid-to-plasma albumin ratio (QAlb) (70, 88, 89)—and EEG biomarkers of anaesthetic brain responsiveness, such as reduced frontal alpha power or increased burst suppression (30, 33, 90). Recent human data also suggest that preoperative cognitive screening alone may not adequately reflect underlying brain vulnerability (91). The proposed conceptual framework linking age-related neuroimmune vulnerability with increased anaesthetic sensitivity is summarised in Figure 1.

**Figure 1:**
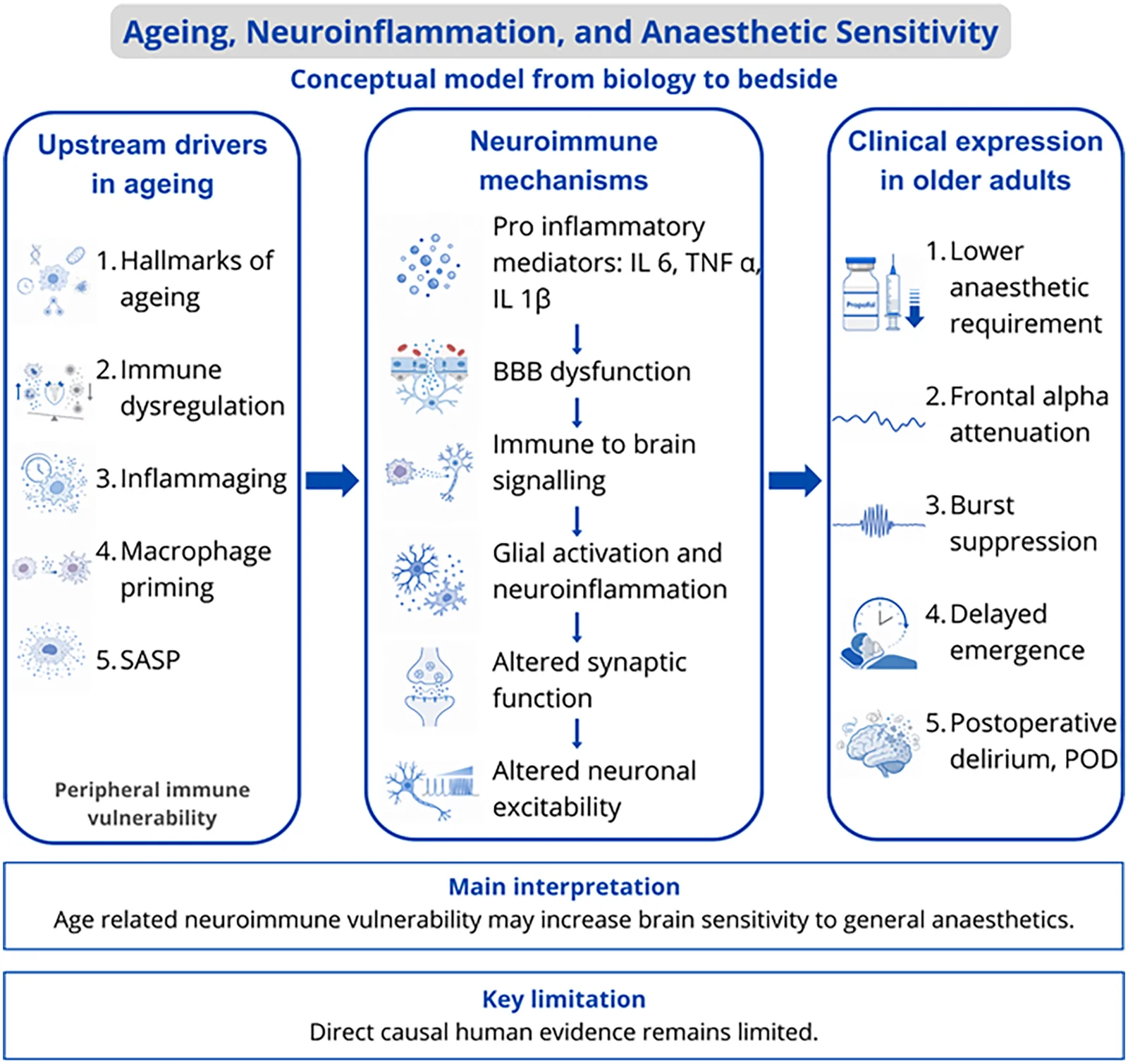
Conceptual model linking age-related neuroimmune vulnerability with increased anaesthetic sensitivity. Ageing is associated with immune dysregulation, inflammaging, peripheral macrophage priming, and senescence-associated secretory phenotypes, which may increase circulating pro-inflammatory mediators, including IL-6, TNF-α, and IL-1β. Through blood–brain barrier dysfunction and immune-to-brain signalling, these systemic signals may promote glial activation, neuroinflammation, and altered synaptic and neuronal function. In older adults, this neuroimmune vulnerability may contribute to lower anaesthetic requirements, attenuated frontal alpha activity, burst suppression, delayed emergence, and increased risk of postoperative delirium. This model is biologically plausible and supported mainly by preclinical and indirect clinical evidence; however, direct causal human evidence remains limited.

## 6 Neuroinflammation and anaesthetic sensitivity

### 6.1 Preclinical evidence linking inflammation to anaesthetic depth

Rodent models have provided important evidence that peripheral inflammation can influence the brain's response to anaesthetic agents. For instance, Cibelli showed that peripheral surgical trauma in mice induces neuroinflammatory changes, microglial activation, and impaired hippocampal synaptic plasticity, supporting the concept that systemic inflammatory insults can alter central neural function (72). More directly, Avramescu et al. showed that inflammation increases neuronal and behavioural sensitivity to general anaesthetics, suggesting that systemic inflammatory states may lower anaesthetic requirements independently of drug metabolism (9). Together, these findings support the view that inflammatory states may increase anaesthetic sensitivity through mechanisms that extend beyond drug metabolism alone.

### 6.2 Mechanisms: CNS excitability, synaptic changes, and EEG alterations

Several mechanisms may account for this inflammation-induced anaesthetic hypersensitivity.

#### 6.2.1 Cytokine-mediated modulation of synaptic function

Pro-inflammatory cytokines such as IL-1β, IL-6, and TNF-α, released by activated macrophages, microglia, or astrocytes, may impair long-term potentiation (LTP) and disrupt glutamatergic and GABAergic signalling, both of which are key targets of anaesthetic drugs (72). These changes may reduce synaptic efficacy and alter neuronal excitability, thereby lowering the amount of anaesthetic required to induce unconsciousness.

#### 6.2.2 Microglial priming and neurotransmission disruption

Aged microglia adopt a primed or sensitized phenotype and respond more robustly to peripheral inflammatory signals, including systemic LPS challenge, with exaggerated cytokine production and neuroinflammatory activation (83, 92). These amplified neuroimmune responses may disrupt neuronal function and impair network synchrony. Although direct evidence linking microglial priming to anaesthetic EEG signatures remains limited, systemic inflammation has been shown to alter EEG-derived measures in mouse models of delirium, supporting the possibility that inflammatory states may modify cortical coordination under anaesthetic or perioperative conditions (93).

#### 6.2.3 Altered thalamocortical dynamics

Thalamocortical circuits are critical for maintaining consciousness and are disrupted by general anaesthetics (94–96). Experimental and translational evidence suggests that inflammatory processes can alter ion channel function, oscillatory bursting, and synaptic communication within the thalamocortical system (97). Together, these observations support the possibility that neuroinflammation may contribute to altered emergence profiles and greater anaesthetic depth for a given drug dose, although direct human evidence remains limited.

#### 6.2.4 Blood–brain barrier dysfunction

Age-related changes such as tight junction disruption, endothelial dysfunction, and increased vesicular transcytosis may contribute to heightened BBB permeability (Figure 2). This may facilitate the passage of peripheral inflammatory mediators into the CNS, amplifying neuroinflammation and further altering neuronal responsiveness to anaesthetic agents (42, 75, 76).

**Figure 2:**
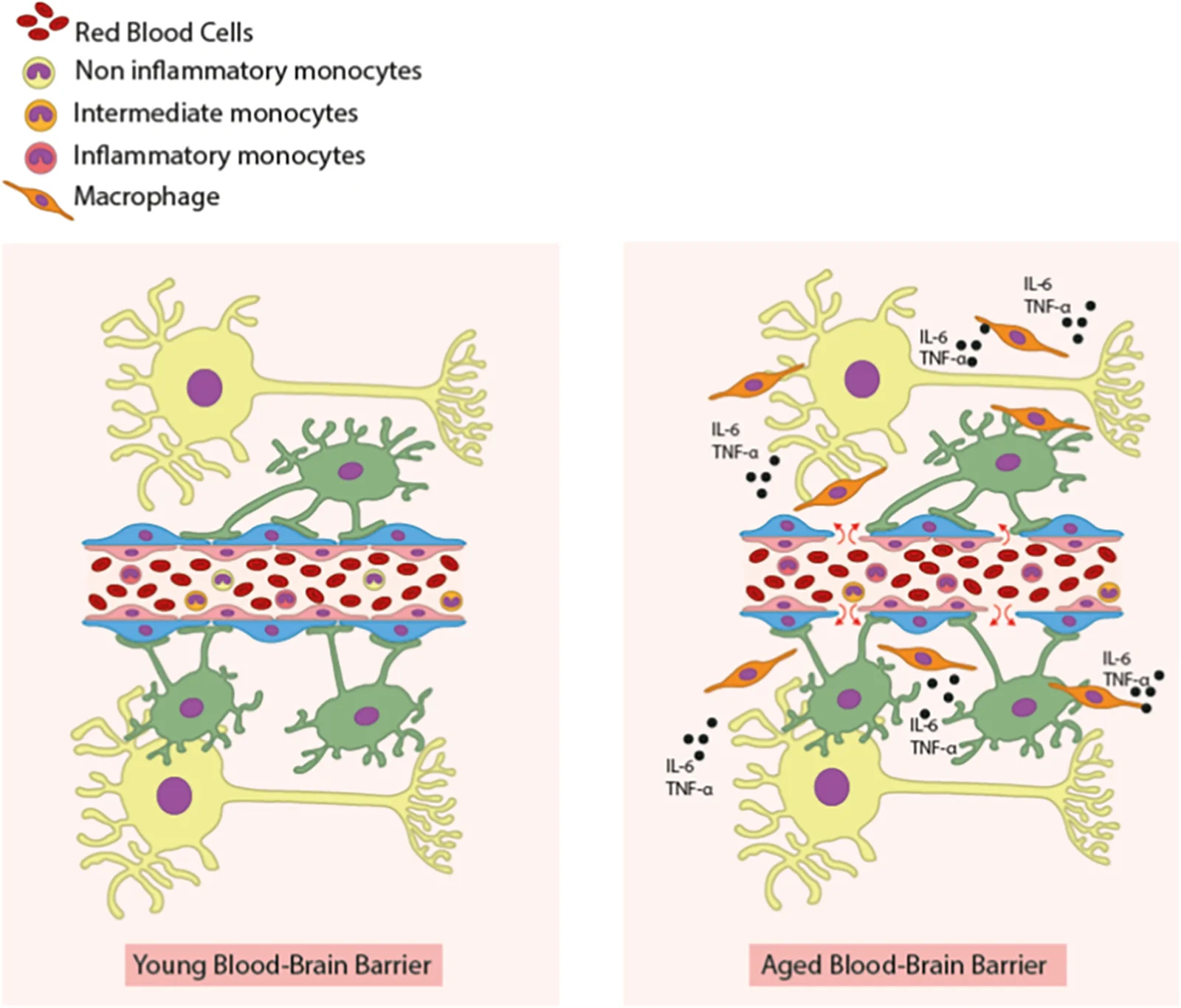
Age-related blood-brain barrier (BBB) hyperpermeability promotes peripheral macrophage infiltration into the brain parenchyma. Schematic representation comparing the structural and functional integrity of the BBB in young (left) and aged (right) brains. In the young brain, a fully functional BBB maintains restricted permeability, preventing the entry of circulating immune cells. Conversely, the aged BBB exhibits increased vascular permeability. This loss of barrier integrity leads to the subsequent infiltration of peripheral blood-derived macrophages into the cerebral tissue, a key driver of age-associated neuroinflammation.

## 7 Clinical evidence, translational gaps, and future directions

These animal model findings offer a biological explanation for clinical observations in older adults. Specifically, older adults often require lower anaesthetic doses, show EEG suppression at standard doses, and appear more susceptible to delayed emergence and postoperative neurocognitive complications, including POD. Neuroinflammation is considered an important pathological contributor to perioperative neurocognitive vulnerability. It is also recognised that patients with EEG markers associated with increased sensitivity to general anaesthetics, such as lower intraoperative alpha power and greater burst suppression, have a higher likelihood of POD (30, 33). Thus, it is plausible to hypothesise that increased sensitivity to general anaesthetic effects and greater perioperative neurocognitive vulnerability may, at least in part, reflect immune-mediated pharmacodynamic changes in neuronal function.

Despite compelling preclinical evidence supporting the role of peripheral inflammation, macrophage activation, and BBB dysfunction in modulating anaesthetic sensitivity, clinical translation remains limited. Most human data derive from observational studies and anaesthetic-depth trials in older surgical patients, in whom low processed EEG values, intraoperative EEG suppression, or suppression occurring at lower volatile anaesthetic concentrations have been associated with postoperative delirium and adverse neurocognitive outcomes (33, 98, 99). In parallel, perioperative studies have linked postoperative delirium with blood–brain barrier dysfunction and neuroinflammatory biomarkers in cerebrospinal fluid, supporting the clinical relevance of immune–brain interactions in vulnerable older patients (88, 89). However, these clinical associations do not establish that immune dysregulation directly alters anaesthetic pharmacodynamics in humans. Definitive causal links between inflammation, BBB dysfunction, and altered anaesthetic sensitivity therefore remain elusive.

Notably, studies evaluating QAlb have suggested increased BBB permeability in older adults, particularly in those who develop POD. In a prospective perioperative cohort, Taylor et al. evaluated perioperative changes in CSF-to-plasma albumin ratio as a fluid biomarker of BBB permeability and found that these changes were associated with delirium incidence and severity, alongside CSF IL-6 and lactate responses (89). The authors also measured IL-6 and TNF-α in cerebrospinal fluid and plasma, reinforcing a possible link between systemic inflammation and BBB disruption. Similarly, Montagne used dynamic contrast-enhanced magnetic resonance imaging to assess BBB integrity in ageing adults with varying cognitive function, showing that APOE4 carriers had increased hippocampal leakage, which correlated with cognitive decline and inflammatory changes (70).

Concurrent elevations in systemic cytokines such as IL-6, TNF-α, and IL-1β have also been associated with POD and with poorer postoperative cognitive trajectories (100, 101). Dillon examined high-sensitivity C-reactive protein (hs-CRP) and IL-6 levels in 560 older adults undergoing major surgery and found that elevated preoperative inflammatory markers predicted higher rates of POD (100). Likewise, Vasunilashorn investigated perioperative changes in TNF-α, IL-10, and IL-6 in 351 older adults, linking postoperative cytokine surges with delirium onset (101).

Recent clinical studies have also shown that older patients may exhibit greater EEG burst suppression at lower anaesthetic doses, potentially reflecting increased brain sensitivity rather than pharmacokinetic variation alone (90). These findings may be consistent with the possibility that inflammation-related alterations in neural excitability and receptor function contribute to a lower threshold for anaesthetic-induced unconsciousness, although this link has not been demonstrated directly in humans. In the ENGAGES trial, EEG-guided anaesthesia reduced burst suppression duration, yet did not significantly affect delirium incidence, suggesting that burst suppression is unlikely to be explained by dosage alone and may instead reflect broader biological vulnerability (90). Similarly, Fritz retrospectively analysed intraoperative EEG suppression in more than 1,000 patients and found that suppression duration was a strong predictor of POD, independent of anaesthetic dose (33).

Furthermore, heterogeneity in patient populations, anaesthetic regimens, and perioperative care complicates the interpretation of interactions between immune signalling and anaesthetic responses. Chronological age alone may inadequately capture the biological heterogeneity and vulnerability of older surgical patients. Frailty reflects a multidimensional loss of physiological and homeostatic reserve and can distinguish individuals with substantially different vulnerability despite similar chronological age (102, 103). Importantly, frailty is closely intertwined with immunosenescence and inflammaging, two interacting features of immune ageing that may contribute to impaired homeostatic resilience and increased susceptibility to physiological stressors (103, 104). These relationships suggest that chronological age and biological vulnerability should not be considered interchangeable, particularly when investigating neuroimmune mechanisms of anaesthetic sensitivity. Future studies should therefore incorporate frailty assessment and, where feasible, complementary measures of biological ageing alongside chronological age to determine whether neuroimmune phenotypes better explain interindividual differences in anaesthetic pharmacodynamics.

Biological sex represents an additional source of heterogeneity in ageing that cannot be captured by chronological age alone. Female and male ageing trajectories differ as a consequence of sex-specific reproductive strategies and life-history transitions, with events such as pregnancy, lactation, and particularly reproductive decline and menopause potentially influencing somatic maintenance and susceptibility to age-related disease (105). These observations suggest that sex may modify the relationship between chronological age, biological vulnerability, and immune ageing rather than acting simply as a demographic covariate. Future studies investigating inflammaging and anaesthetic pharmacodynamics should therefore incorporate sex-stratified analyses and, particularly in women, consider reproductive ageing as a potential modifier of neuroimmune and anaesthetic phenotypes (105). More broadly, these sex-specific trajectories reinforce the need to conceptualise ageing as a heterogeneous biological process rather than defining vulnerability solely by chronological age.

The lack of standardised immunophenotyping, inconsistency in defining anaesthetic sensitivity, for example by MAC, BIS, or EEG alpha power, and limited integration of multimodal data, including immune, EEG, and clinical outcomes, continue to restrict mechanistic insight.

Prospective studies incorporating simultaneous immunological profiling, neurophysiological monitoring, and pharmacokinetic and pharmacodynamic modelling are urgently needed. Such integrative approaches could help disentangle whether inflammaging-related pathways directly modulate anaesthetic brain sensitivity or instead reflect a broader background vulnerability. Additionally, interventional studies targeting inflammation, such as perioperative anti-cytokine therapies, senolytics, or strategies aimed at preserving BBB integrity, may serve as valuable probes to test mechanistic hypotheses in human subjects.

Ultimately, bridging preclinical and clinical domains will require multidisciplinary frameworks combining anaesthesiology, immunology, neurobiology, and geriatrics to develop more personalised anaesthetic strategies for ageing populations.

## 8 Perioperative interventions and potential therapeutic targets

### 8.1 Before surgery

Preoperative management should focus on identifying biological and neurocognitive vulnerability rather than relying on chronological age alone. Frailty, cognitive status, comorbid inflammatory conditions, and potentially inflammatory biomarkers may help identify patients at greater risk of altered anaesthetic responses, although immune biomarkers are not currently validated for anaesthetic dose selection (35). Targeted anti-inflammatory, anti-cytokine, or senolytic strategies remain investigational.

### 8.2 During surgery

Intraoperative management should aim to individualise anaesthetic exposure according to brain responsiveness. Age-adjusted dosing and EEG monitoring may help detect excessive cortical depression and guide titration in older or frail patients (14). In ENGAGES, EEG-guided anaesthesia reduced anaesthetic exposure and EEG suppression but did not reduce postoperative delirium, indicating that these outcomes should not be considered equivalent therapeutic targets (90). Future studies should determine whether neuroimmune phenotypes require different concentration–effect targets rather than simply lower age-based doses.

### 8.3 After surgery

Postoperative care should minimise additional physiological and inflammatory stress while facilitating early detection of neurological dysfunction. Multicomponent strategies including orientation, sleep preservation, mobilisation, hydration, sensory support, and adequate pain control are recommended in patients at risk of delirium (35). Whether targeted modulation of cytokines, complement signalling, cellular senescence, or BBB dysfunction improves neurophysiological or cognitive recovery remains unknown.

## 9 Conclusion

Anaesthetic care in older adults sits at the intersection of ageing biology and neuroimmune physiology. This review emphasises that peripheral macrophages are central actors at that interface: through inflammaging and the senescence-associated secretory phenotype, they may promote cytokine-mediated signalling, including IL-6, TNF-α, and IL-1β that can shift CNS excitability, interact with anaesthetic targets, and, when the BBB is vulnerable, amplify neuroinflammation in the perioperative period. Clinically, these processes may help explain why many older patients show greater anaesthetic sensitivity, attenuated frontal alpha power, and more pronounced burst suppression at doses that are otherwise standard, and they may contribute to broader perioperative brain vulnerability, including POD and delayed recovery.

For clinical practice, the implication is not simply “less drug”, but individualised titration informed by EEG patterns, frailty, cognitive status, and, where feasible, inflammatory profiling, while recognising that current human evidence linking specific immune markers to real-time dosing decisions remains limited. For research, the priority is integrative perioperative studies that couple monocyte and macrophage phenotyping and BBB metrics with EEG and pharmacodynamic modelling, as well as mechanism-guided interventions, including immune or endothelial modulation, tested against neurocognitive outcomes.

Framed this way, inflammation, anaesthetic sensitivity, and ageing are not parallel concerns but parts of a shared pathway, with peripheral macrophages emerging as both candidate biomarkers and potential mechanistic targets for protecting the vulnerable surgical brain.
